# Efficacy and Cost-Effectiveness of Laparoscopic Transversus Abdominis Plane (TAP) Block in Laparoscopic Cholecystectomy: A Comparison With the Non-TAP Group

**DOI:** 10.7759/cureus.32038

**Published:** 2022-11-30

**Authors:** Pallavi Nair, Chinmaya R Behera, Rajat K Patra, Nithya Shekar, Lakshmi S Rao, Pransingh Pujari, Bandita Panda, Amaresh Mishra

**Affiliations:** 1 Department of Surgery, Kalinga Institute of Medical Sciences, Bhubaneswar, IND; 2 Department of Research and Development, Kalinga Institute of Medical Sciences, Bhubaneswar, IND

**Keywords:** transversus abdominis plane (tap), transversus abdominis plane, usg-tap block, pain assessment, laparoscopic cholecystectomy, non-tap block, transversus abdominis plane block (tap block)

## Abstract

Background: Postoperative pain caused by laparoscopic cholecystectomy can be controlled by different methods. The study aimed to observe the efficacy of laparoscopic transversus abdominis plane (TAP) block in laparoscopic cholecystectomy and to analyze the cost-effectiveness of the procedure in comparison to the non-TAP method.

Methods: In this double-blinded randomized clinical trial, the subjects who had come for cholecystectomy were randomly divided into two groups (n = 43 in each group). Group A received laparoscopy-guided subcostal TAP block bilaterally (0.25% bupivacaine, 20 ml each) along with parenteral analgesics (100 mg tramadol injection in 100 ml normal saline IV) SOS as rescue analgesia, and group B received parenteral analgesics (injection paracetamol 1 gm IV) eight hourly and injection tramadol 100 mg in 100 ml normal saline IV SOS as rescue analgesia.

Result: A bulge was visualized by the surgeon through a laparoscope as a signature view for confirming the placement of local anesthetic in TAP. Based on the Visual Analog Scale (VAS) for assessment of postoperative pain and the Numeric Rating Scale (NRS) for assessment of pain at 30 minutes, four hours, eight hours, 12 hours, and 24 hours postoperatively, patients of both groups were assessed. According to the VAS, the pain assessment was better in the TAP block group at 30 minutes post-surgery than in the non-TAP group. As a primary outcome, 37% of TAP block group cases were recovered without any rescue analgesia. VAS score revealed a significant difference in postoperative nausea and vomiting (PONV) among the TAP block and non-TAP groups. PONV at four hours, eight hours, and 12 hours showed significantly lesser incidences in the TAP group as compared to the non-TAP group (p-value: 0.015, 0.028, and 0.055, respectively).

Conclusion: The cost-effectiveness of the TAP block method is 20 times lesser than the non-TAP method. Thus, a laparoscopic-guided TAP block could offer better postoperative analgesia at a low cost with a similar advantage to a USG-guided TAP block.

## Introduction

Laparoscopic cholecystectomy is a minimally invasive procedure, which involves parietal, visceral, incisional, and referred postoperative pain [1,2]. Postoperative pain in laparoscopic cholecystectomy is mainly from the port sites, but to a lesser extent from peritoneal stretching due to pneumoperitoneum and the presence of residual gas in the peritoneal cavity. Analgesics are being used not only to bear the pain and stress of surgery but also to help in early ambulation and limit complications like postoperative lung atelectasis, hypoxemia, and deep vein thrombosis, hence adequate postoperative pain management is the key factor to achieving a better surgical outcome. The present concept for pain management is the multimodal approach. In the 1980s, a technique involving multiple injections of local anesthetic in the abdominal wall was used [3] The transversus abdominis plane (TAP) block procedure on the abdomen mainly acts on the myocutaneous nerves of the anterior abdominal muscle compartment, parietal region, and incision site pain, which was in TAP block using single needle puncture via the lumbar triangle of Petit [4]. Recently, ultrasound-guided TAP block is being used for improved results [5,6]. The other advantages of TAP block are the opioids sparing effects, reduction in pain scores, good patient compliance, and overall comfort [7,8]. The laparoscopic TAP block technique is applied under the surgeon’s vision, which is confirmed by the intra-peritoneal bulge, visualized using a laparoscope. TAP block is the anatomical space present between the transversus abdominis muscle and the internal oblique muscle [9,10]. In this neurovascular fascial plane, a bolus dose of anesthetic agent is injected, which leads to blockage of the T7-11 intercostal nerve’s dermatomal afferents, T12 subcostal nerve, ilioinguinal nerve, iliohypogastric nerves, and L1-3 nerves cutaneous branches [11,12]. The anatomical variations in the nerves entering and leaving this neurovascular plane are possible in a few patients, which may lead to the failure of the block. In laparoscopic cholecystectomy, four ports are inserted and made at the supra-umbilical region (10 mm), epigastric region (10 mm), right anterior axillary (5 mm), and right midclavicular line (5 mm). The sensation of pain from abdominal skin muscle and parietal peritoneum is carried by intercostal nerves (T7-T11), subcostal nerve (T12), cutaneous branches of L1-3 nerves, iliohypogastric nerve, and ilioinguinal nerve (L1). The procedure of depositing local anesthetic agents into the TAP can block the above spinal nerves. In laparoscopic cholecystectomy cases, TAP block is commonly given by classical or posterior approach [6], which provides analgesia from T7 to T10 dermatome. The subcostal TAP block technique given bilaterally can cause T6 dermatome blockage, which is the area of the epigastric port in laparoscopic cholecystectomy [13]. Laparoscopic-assisted TAP block is faster and more efficacious, which will have a very important role in centers where the USG machine is not available in operating rooms for providing ultrasound-guided block by the anesthesia team. The present study aimed to evaluate the effectiveness as well as safety of TAP block in laparoscopic cholecystectomy for pain control postoperatively and to compare with an additional analgesics requirement in patients who were with or without TAP block during laparoscopic cholecystectomy.

## Materials and methods

A prospective study with a parallel randomized trial was conducted from September 2019 to September 2021 in a tertiary care center. The patients aged between 20 and 80 years who were undergoing laparoscopic cholecystectomy at our hospital (American Society of Anesthesiologists (ASA) grade I and II) were included in the study after signing the consent form. Ethical approval was obtained from the Institutional Ethics Committee, Kalinga Institute of Medical Sciences, Bhubaneswar (KIMS/RPC/119). Patients having acute/chronic/acute chronic calculous cholecystitis were diagnosed by ultrasonographic evaluation of the abdomen. Demographic features like age, gender, BMI, history of previous abdominal surgeries and history of comorbidities, previous hospitalization, acute cholecystitis/acute pancreatitis, and analysis of ultrasonographic evidence were recorded. Based on the duration of symptoms, associated comorbidities, and ultrasonographic evidence, patients were planned for laparoscopic cholecystectomy. For comparison, participants were divided into two groups. Randomization was done by a computerized method. One group, group A, received laparoscopy guided subcostal TAP block bilaterally (0.25% bupivacaine, 20 ml each) along with parenteral analgesics (100 mg tramadol injection in 100 ml normal saline IV) SOS as rescue analgesia, and the second group, group B, received parenteral analgesics (injection paracetamol 1 gm IV) eight hourly and injection tramadol 100 mg in 100 ml normal saline IV SOS as rescue analgesia. After completion of the laparoscopic cholecystectomy, a TAP block was applied to one group (group A). A 22G needle was used to puncture at the right and left of the subcostal region, lateral to the rectus. The needle localization was done under the laparoscopic vision and the tip of the needle was positioned at the TAP space, which was between the internal oblique and the transversus abdominis muscle. A preventive aspiration was done, and 0.25% bupivacaine, 20 ml each, was injected on both sides. A bulge was noted by the surgeon through laparoscope due to the separation of the transversus abdominis muscle and the internal oblique muscle by the drug volume; thus, confirming the placement of local anesthetic in TAP.

**Figure 1:**
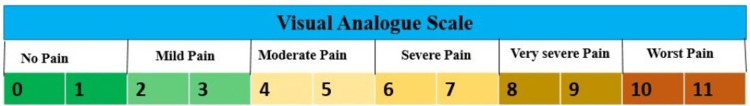
Visual Analog Scale (VAS) for the assessment of postoperative pain VAS 0-1: no pain; VAS 2-3: mild pain; VAS 4-5: moderate pain; VAS 6-7: severe pain; VAS 8-9: very severe pain; VAS 10-11: worst pain.

The patients were interviewed and monitored for 30 minutes, four hours, eight hours, 12 hours, and 24 hours postoperatively. Patients were assessed on their Visual Analog Scale (VAS) score, which is a numerical rating score (Figure 1). Injection of tramadol 100 mg in 100 ml normal saline was provided as rescue analgesics on patients’ demand if they had pain postoperatively, despite drugs received in TAP and non-TAP groups.

Patients were compared based on the following parameters: patient’s age, gender, duration of operation, Numerical Rating Scale (NRS) score for assessment of pain at 30 minutes, four hours, eight hours, 12 hours, and 24 hours postoperatively, the requirement of analgesia post-surgery in both groups, duration of hospital stay, and the patients who will be willing to comply with the study group.

Patients with gallbladder empyema, previous upper abdominal surgery with intra-abdominal adhesions, other conditions that will interfere with the biliary tract surgery, hematological and bleeding disorders, a provisional diagnosis of gallbladder cancer, incomplete data, and ASA grade more than II were excluded from the study.

As per the power calculation, the sample size was 43 in each group, i.e., group A (patients receiving TAP block) and group B (patients receiving parenteral analgesics).

The detailed history and clinical examination of the patient were recorded. Clinical examinations such as complete blood count (CBC), random blood sugar, serum urea, creatinine, electrolytes, viral marker, liver function tests (HIV, hepatitis B surface antigen, hepatitis C virus, prothrombin time, and international normalized ratio), ultrasonography of the abdomen and pelvis, and specific investigations or procedures including contrast-enhanced CT scan, magnetic resonance cholangiopancreatography (MRCP), and endoscopic retrograde cholangiopancreatography (ERCP) were conducted for the study participants.

With preoperative informed consent about the study methods, patients were included in the study. Statistical parameters like mean and standard deviation (SD) were applied for continuous data and frequency by percentage was used for categorical data. For comparing categorical data between TAP and non-TAP groups, we used an independent t-test and chi-square test or Fisher's exact test. Repeated measure ANOVA was used to check the effect of time and between groups for VAS and pulse rate (PR). Statistical software IBM SPSS version 23.0 (IBM Corp., Armonk, NY) was used. A p-value less than or equal to 0.05 was considered statistically significant.

## Results

Demographic features

**Figure 2:**
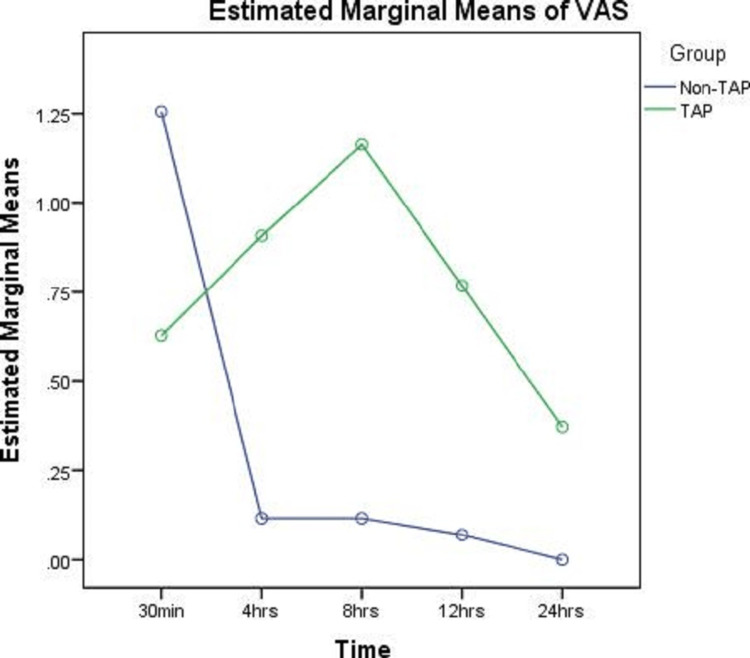
Comparative assessment of pain based on VAS between TAP and non-TAP group VAS: Visual Analog Scale; TAP: transversus abdominis plane.

In the present study, a total of 86 patients were randomly included in an age group of 20-80 years. Out of 86 patients, 62 patients were female and 24 were male. The mean weight ranged from 64 to 69.86 kg. Assessment of postoperative pain was done based on the VAS in two groups. In the TAP block group, the mean VAS pain assessment was 0.63 as observed 30 minutes post-surgery, which was gradually increased till eight hours to 1.16 and then subsequently came down to 0.37. Compared with this, the non-TAP group showed a classical pain pattern, where the mean VAS pain assessment was 1.26 at 30 minutes post-surgery, which subsequently decreased at four hours post-surgery, and gradually by the end of 24 hours, the pain was negligible. In the VAS, there is a significant difference in pain among the TAP and non-TAP groups at 30 minutes post-surgery (Figure 2).

VAS rescue analgesia

**Figure 3:**
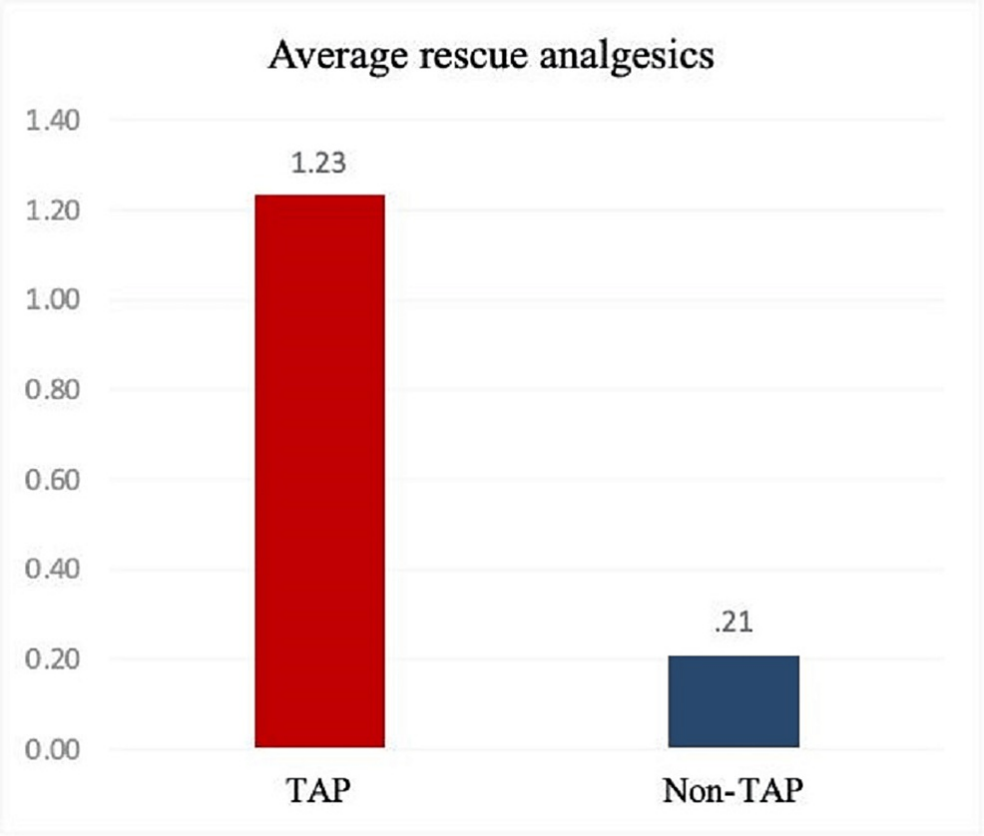
Average post-surgery rescue analgesics requirement in both TAP block and non-TAP group TAP: transversus abdominis plane.

Out of 43 patients in the non-TAP group, 38 patients had not received any rescue analgesia, and two patients each required one and two rescue analgesia, respectively, whereas, in the TAP block group (n = 43), 24 patients needed two rescue analgesics and 16 patients had not received any rescue analgesia (Figure 3).

The pain assessment after rescue analgesics in the TAP block group was 1.23 ± 1.00, while in the non-TAP group, it was found to be 0.21 ± 0.64 (Figure 3). Hence, a significant difference was found in the requirement of rescue analgesics post-surgery between the two groups and the p-value was <0.001, which is statistically significant.

Duration of surgery

The mean duration of the surgery in the TAP block group was 88.90 minutes, while that of the non-TAP group was 93.60 minutes. Hence, there is no significant difference in the duration of surgery between the two groups. The p-value was 0.108, which is statistically insignificant.

The average duration of giving laparoscopic TAP block was around three to five minutes only. The drugs were prepared by the scrub nurse during the time of surgery. There was no extra time required to give a TAP block.

Postoperative nausea and vomiting

In the first 30 minutes, both TAP block group and non-TAP group patients did not have a significant difference in the incidence of postoperative nausea and vomiting (PONV); however, in four hours, eight hours, and 12 hours of monitoring, the majority of the patients in the TAP group had no nausea and vomiting as compared to the non-TAP group (Table 1). The data in four hours and eight hours were statistically significant as p < 0.05, and the data in 12 hours was not significant. In 24 hours of monitoring, no patient in the TAP block had nausea and vomiting, whereas one patient had moderate and severe nausea in the non-TAP block.

**Table 1:** Postoperative nausea and vomiting (PONV) assessment in percentage based on the Numeric Rating Scale (NRS) TAP: transversus abdominis plane.

PONV	Category		Non-TAP (%) (n = 43)	TAP (%) (n = 43)	P-value
30 minutes	0	No nausea	83.72	86.05	1
	1	Mild nausea	13.95	13.95	
	2	Moderate nausea	2.33	0.00	
	3	Severe nausea	0.00	0.00	
	4	Emesis	0.00	0.00	
4 hours	0	No nausea	74.42	95.35	0.015
	1	Mild nausea	9.30	4.65	
	2	Moderate nausea	13.95	0.00	
	3	Severe nausea	2.33	0.00	
	4	Emesis	0.00	0.00	
8 hours	0	No nausea	79.07	97.67	0.028
	1	Mild nausea	4.65	2.33	
	2	Moderate nausea	9.30	0.00	
	3	Severe nausea	4.65	0.00	
	4	Emesis	2.33	0.00	
12 hours	0	No nausea	88.37	100.00	0.055
	1	Mild nausea	2.33	0.00	
	2	Moderate nausea	4.65	0.00	
	3	Severe nausea	4.65	0.00	
	4	Emesis	0.00	0.00	
24 hours	0	No nausea	95.35	100.00	0.494
	1	Mild nausea	0.00	0.00	
	2	Moderate nausea	2.33	0.00	
	3	Severe nausea	2.33	0.00	
	4	Emesis	0.00	0.00	

Duration of hospital stay post-surgery

Both groups had no significant difference in duration of hospital stay and were found in the average postoperative hospital stay of two days with a p-value of 0.067.

Cost-effectiveness

In the TAP block group, 0.5% bupivacaine 20 ml was used with 20 ml of distilled water (to obtain 40 ml of 0.25% bupivacaine), which cost Indian rupee (INR) 20. Injection of tramadol 100 mg in 100 ml normal saline was given as a rescue analgesic, which cost INR 21. The average number of times rescue analgesia had to be provided to a patient in the TAP block was 1.23. Hence, the average cost per patient in the TAP block was INR 45.83 (i.e., 20 + 1.23 x 21).

In the non-TAP group, an injection of paracetamol 1 gm was given thrice daily, which cost INR 300 x 3 = INR 900. Injection of tramadol 100 mg in 100 ml normal saline was given as rescue analgesics, which cost INR 21. The average number of times rescue analgesia had to be provided to a patient in the TAP block was 0.21. Hence, the average cost per patient in the non-TAP block was INR 904.41 (i.e., 900 + 0.21 x 21).

Thus, in this study, it was found that the average net expenditure for postoperative pain relief was comparatively more in the non-TAP block group than the TAP block group (20 times in the non-TAP group as compared to TAP group), with a p-value of 0.0001, which is statistically significant.

## Discussion

Reduced postoperative pain and quick recovery are the major advantages of minimally invasive surgery [1]. For pain control after surgery, different analgesic treatments are mostly used. The TAP block technique was first reported by Rafi, and it offers effective pain control after surgery and allows the surgeon to do it under direct vision. Its other benefits are a reduction in the intensity of pain, a decrease in side effects from analgesics, enhance post-surgery recovery, and reduced cost of analgesia [4].

The present study denotes that 0.25% bupivacaine as laparoscopic TAP block was effective in 37.20% of patients undergoing laparoscopic cholecystectomy and provides effective postoperative analgesia, with no requirement for rescue analgesia, as the patients did not have complaints of pain postoperatively. Overall, in our study, the TAP block alone is found to be an effective method in the initial period of the first four to eight hours along with a definitive need for rescue analgesia after eight hours. There could be partial block in some cases, possibly due to a lack of administration of the local anesthetic drug in the proper TAP region. The clear bulge visualized in the peritoneum through the laparoscope, which was taken as a marker, could also occur if the drug is administered below the transversus abdominis muscle (in the pre-peritoneal space).

Postoperative analgesia, VAS score, postoperative nausea and vomiting, and other side effects were compared in groups A and B. Our results are correlating with the studies done by McDonnell et al. [6] in cases of abdominal surgery and Carney et al. [13] in cases of open appendicectomy. Carney et al. and Sharma et al. justified the use of TAP block in cases of total abdominal hysterectomy patients who had shown significantly reduced postoperative pain till 48 hours postoperative period [14-16]. A meta-analysis study explained that TAP block reduced postoperative consumption of opioid agents, and thereby played an important role in analgesia regimen. The increased time of analgesic effect after a single injection of TAP block is due to the TAP being relatively less vascularized, and thus drug clearance is slower [15]. There is a possibility of incomplete pain management, though TAP block is applied, which can be explained by failure in the technical aspect or due to involvement of the visceral pain component, which is not managed by TAP block. All available local and regional anesthetic methods also have a failure rate of 5-20% inherently, which is based on the operator’s skill [16].

Laparoscopic cholecystectomy is done under general anesthesia and thus is an excellent opportunity to perform TAP block intraoperatively by the laparoscopic method, which avoids operating room time delays as compared to USG-guided TAP block. We carried out a TAP block at the end of the surgery.

The advantage of a TAP block in giving immediate postoperative analgesia in 30 minutes is found in our study, which is explained by a lesser VAS score as compared to the non-TAP block. There is a need for additional rescue analgesics for patients in the TAP group after four hours as there was a need for rescue analgesia in our patients. Two patients received one rescue analgesic, 24 patients received two rescue analgesics, and one patient received three rescue analgesics from four hours post-surgery till 24 hours of assessment.

In our study, TAP group patients had excellent pain relief in 30 minutes postoperative assessment than the non-TAP group. However, as time progressed, better pain relief was noted in the non-TAP group as compared to the TAP group. This may be due to inadequate drug instillation in the TAP region (which is between the internal oblique muscle and transversus abdominis muscle of the anterior abdominal wall) where cutaneous nerves needed to be blocked, but instillation of drug mainly in the transversus abdominis muscle region would not result in the desired effect. Laparoscopic TAP block with a visualization of the bulge as a marker for installation of the drug in transversus abdominis muscle pain in patients undergoing laparoscopic cholecystectomy surgery is an alternative mode of pain management in the absence of ultrasound-guided TAP block.

Opioids associated with nausea-vomiting, pruritus, and respiratory depression were managed by injection of paracetamol as analgesics in the non-TAP group, and injection of tramadol was used as rescue analgesia in both groups.

In laparoscopic cholecystectomy, bilateral TAP block gives very good pain management to the skin as well as anterior abdominal wall muscles. The bupivacaine laparoscopic TAP block group showed good satisfaction in 16 patients out of 43 patients and was able to mobilize without any pain or discomfort as compared to the non-TAP group. The efficacy of the TAP block was better in 37% of patients in the TAP block group. This result is very well supported by the findings of McDonnell et al. [6], which explain prolonged pain relief by TAP block till or beyond 24 hours, which is due to relatively less vascularization of the TAP plane and thus slowed drug clearance. The TAP group and non-TAP group showed a similar pattern with respect to heart rate and blood pressure and no statistical significance was noted, which could be explained by no pain-mediated sympathetic stimulation (stress response) because TAP block was as good as parenteral (tramadol) analgesics given 8th hourly.

In our study, the incidence of PONV was very much less for both groups A and B, as we used a weaker opioid (i.e., tramadol) as compared to morphine.

The process of blinding was challenging in the study. Investigators did not access the level of sensory blockade level and assessed only VAS scores. In our study, peritoneal and visceral punctures related to TAP block were not encountered, which has been reported in the literature by investigators before. Farooq and Carey [16] reported a patient having liver trauma who underwent a TAP block while a blunt regional anesthesia needle was used.

**Figure 4:**
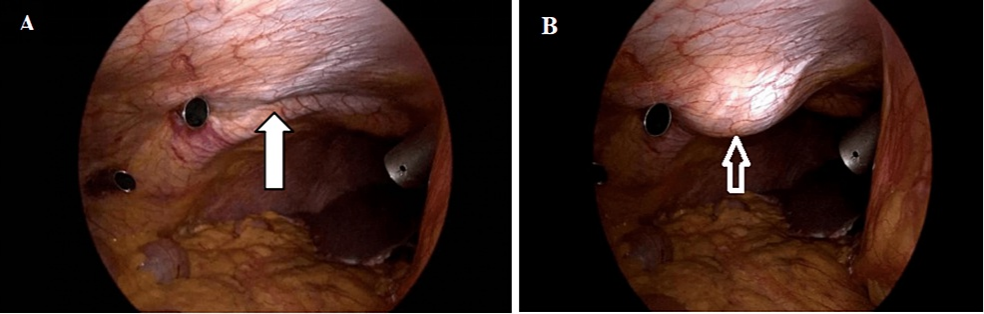
Intraoperative bulge due to instillation of the agent through the laparoscopic transversus abdominis plane

As compared with other studies, the safety of laparoscopic TAP block is established in our study also, as no local or systemic toxicity was encountered, and hence this technique should remain one of the main pain management methods. The laparoscopic TAP block method with a bulge visualized by the surgeon through a laparoscope as a signature view for confirming the placement of local anesthetic in TAP is reliable and efficacious if used along with additional rescue analgesics (Figure 4).

Limitations

The limitation of the study is a need for a larger randomized, multi-institutional study to establish the optimal benefits, efficacy, as well as technical aspect of giving bilateral subcostal laparoscopic TAP block in patients undergoing laparoscopic cholecystectomy, as identification of plane remains a challenge, and also the need for another revised effective dose may be considered.

## Conclusions

In laparoscopic cholecystectomy, postoperative pain is mainly from the port sites and peritoneal stretching due to pneumoperitoneum, and the presence of residual gas in the peritoneal cavity causes irritation. The abdominal wall pain can be controlled by TAP block, but the visceral pain, which is carried by sympathetic fibers, will need systemic analgesics for complete pain management. Hence, a multimodal approach is the best method for effective pain control in laparoscopic cholecystectomy, as the analgesics will have a synergistic effect on each other.

The laparoscopic TAP block is a regional analgesic technique given by the surgeon under vision intraoperatively for pain management. A bulge is noted by the surgeon through laparoscope as a signature view of the TAP block for confirming the placement of local anesthetic in TAP. The present study was successful in 37% of cases without any rescue analgesia, and in rest, till eight hours after surgery, the pain management was significantly better than the non-TAP method, which establishes the effectiveness of laparoscopic TAP block. We find laparoscopic TAP block to be a safe procedure with no local or systemic side effects. In a laparoscopic TAP block, only three to five minutes of extra time is needed for giving the drug, which is not a time-consuming procedure. This is cost-effective (20 times lesser cost) as compared to routine non-TAP group analgesic paracetamol infusion. In comparison to USG-guided TAP block, intraoperative laparoscopic TAP block avoids time delays in operation theater and the use of extra equipment like a USG machine.
